# Hostility and Cellular Aging in Men from the Whitehall II Cohort

**DOI:** 10.1016/j.biopsych.2011.08.020

**Published:** 2012-05-01

**Authors:** Lena Brydon, Jue Lin, Lee Butcher, Mark Hamer, Jorge D. Erusalimsky, Elizabeth H. Blackburn, Andrew Steptoe

**Affiliations:** a Department of Epidemiology and Public Health, University College London, London, United Kingdom; b Cardiff School of Health Sciences, University of Wales Institute, Cardiff, United Kingdom; c Department of Biochemistry and Biophysics, University of California, San Francisco, California

**Keywords:** Aging, gender, hostility, psychological stress, telomerase activity, telomere length

## Abstract

**Background:**

Hostility is associated with a significantly increased risk of age-related disease and mortality, yet the pathophysiological mechanisms involved remain unclear. Here we investigated the hypothesis that hostility might impact health by promoting cellular aging.

**Methods:**

We tested the relationship between cynical hostility and two known markers of cellular aging, leukocyte telomere length (TL) and leukocyte telomerase activity (TA), in 434 men and women from the Whitehall II cohort.

**Results:**

High-hostile men had significantly shorter leukocyte TL than their low-hostile counterparts. They also had elevated leukocyte TA, with a significantly increased likelihood of having both short TL and high TA, compared with low-hostile individuals.

**Conclusions:**

Because telomerase is known to counteract telomere shortening by synthesizing telomeric DNA repeats, particularly in the context of shortened telomeres, heightened TA might represent a compensatory response in high-hostile individuals. The relationship between hostility and disease is stronger in men than in women, and men generally have a shorter life expectancy than women. Our findings suggest that telomere attrition might represent a novel mechanism mediating the detrimental effects of hostility on men's health.

Hostility, an enduring personality trait characterized by “a suspicious, mistrustful attitude toward interpersonal relationships and the wider environment,” is associated with an increased risk of age-related disease and all-cause mortality in humans (1–5). Although this relationship has been known to exist for many years, the biological mechanisms linking hostility and health remain poorly understood.

One biological process increasingly implicated in the association between psychological parameters, pathophysiological processes, and aging is telomere shortening (6–9). Telomeres are ribonucleoprotein complexes that cap and protect chromosome ends. Human telomeric DNA, consisting of tandem hexanucleotide repeats of the sequence TTAGGG, naturally shortens during somatic cell division and as a result of oxidative damage (10). Shortening below a specific threshold length results in telomere dysfunction, leading to genomic instability, apoptosis and/or cell senescence, and has thus been implicated in the loss of tissue homeostasis, organ failure and organismal aging (10,11). A critical determinant of telomere length (TL) is the cellular enzyme telomerase, which counteracts telomere shortening by adding TTAGGG repeats onto the 3' ends of telomeric DNA, thus promoting genomic stability and cell longevity (10,11). Because a short telomere stimulates telomerase action on that telomere, thereby elongating it, and cells with short telomeres can be stabilized by the presence of excess telomerase (12), TL and telomerase form an intricately interdependent dynamic system. Both shortened leukocyte TL and altered leukocyte telomerase activity (TA) are associated with progression of age-related diseases—in particular, cardiovascular disease and cancer—as well as their risk factors, including obesity, diabetes, hypertension, and smoking (11,13–19), and several reports have related shorter leukocyte TL to early mortality (13,16,19,20).

Chronic psychological stress, a factor linked to dysregulated immune function and premature aging (21), has also been related to shorter leukocyte TL in a number of recent studies. High levels of perceived stress were associated with shorter leukocyte TL in healthy premenopausal women (6) and in women with a sibling diagnosed with breast cancer (7). Similarly, primary caregivers of Alzheimer's disease patients were found to have shorter leukocyte TL than age- and gender-matched control subjects (8). In addition, chronic stress has been related to both dampened and, paradoxically, elevated leukocyte TA in caregivers, compared with low-stress control subjects (6,8,9).

Importantly, not all individuals exposed to stress age prematurely, and individual differences in perceived stress, vulnerability, and emotional stress responses likely influence the physiological outcome of stress (21,22). Hostile individuals tend to perceive daily life experiences as more threatening, report higher levels of interpersonal conflict and lower social support, and adopt inefficient coping strategies when faced with stressful situations (2). Accordingly, they often display heightened or prolonged physiological responses to acute emotional stressors (2,4,23,24). The relationship between hostility and heightened stress reactivity is particularly apparent in men (23), and evidence suggests that the detrimental effect of hostility on physical health is greater in men than in women (5). Furthermore, previous studies have shown that hostile individuals are more prone to risky health behaviors and their consequences, including increased smoking and alcohol consumption and greater adiposity (25,26). We predicted that hostility might increase disease susceptibility in humans, particularly men, by promoting stress-related changes in TL and TA in leukocytes, and set out to test these hypotheses in a sample of healthy men and women.

## Methods and Materials

### Recruitment and Eligibility Criteria

Participants were a subsample of the Whitehall II cohort, recruited during 2006–2008 as part of the “Heart Scan study” investigating psychosocial, demographic, and biological risk factors for coronary artery calcification (27,28). They were screened to ensure that they had no history or objective signs of coronary heart disease and no previous diagnosis or treatment for hypertension, diabetes, inflammatory diseases, or allergies. Volunteers were of white European origin, and 56.5% were in full-time employment. Socioeconomic status was defined by most recent or current employment grade within the British civil service. Selection was stratified to include adequate representation of higher (grades 7 to 1), intermediate (higher and senior executive office), and lower (administrative assistant, administrative officer, and executive office) grades. Participants were selected at random from each grade and gender category, on the basis of the aforementioned screening criteria. The sample of 543 included 294 men and 249 women, 53–76 years of age. Measurement of TL did not commence immediately, and there was some loss of TA data due to sample nonviability. Thus 434 participants contributed to TL analyses, and 416 contributed to TA analyses. All participants gave written consent, and the study was approved by the University College London Hospital Committee on the Ethics of Human Research.

### Hostility Measure

Cynical hostility was measured by 10 items from the Cook Medley Hostility Scale (CMHS) (1). The CMHS is a widely used self-reported measure of hostility, assessing cynical, mistrustful attitudes toward others and, to a lesser extent, the propensity of an individual for aggressive responding and experiencing hostile affects. It is reported to have good psychometric properties, including adequate internal validity, good test-retest reliability, and construct validity (29). The 10 items include statements such as “it is safer to trust nobody” and “most people make friends because friends are likely to be useful to them,” and were scored with a binary (true/false) format. Total scores could range from 0 to 10, with higher scores indicating greater hostility. The Cronbach α in this sample was .77.

### Isolation of Peripheral Blood Mononuclear Cells

Peripheral blood mononuclear cells (PBMCs) were isolated from whole blood (20 mL) by density gradient centrifugation on Ficoll Paque Plus (GE Healthcare, Buckinghamshire, United Kingdom) and then stored at −80°C in RPMI-1640 with 10% dimethyl sulfoxide and 20% fetal bovine serum, before analysis.

### Measurement of Leukocyte TL

Genomic DNA was extracted from PBMCs in a QIAcube workstation with the QIAamp DNA blood mini kit (Qiagen, Crawley, United Kingdom) according to instructions of the manufacturer and stored in 10 mmol/L Tris-hydrochloric acid, .5 mmol/L ethylenediamine tetraacetate, pH 9.0 at −20°C. Relative mean TL was measured in triplicate by a monochrome multiplex quantitative real-time polymerase chain reaction assay with a Bio-Rad CFX96 Real-Time PCR Detection System (Bio-Rad, Hemel Hempstead, United Kingdom), as previously described (30). Polymerase chain reactions were carried out in a final volume of 25 μL containing 20 ng of sample DNA diluted in 4 μL of pure water, 12.5 μL of QuantiFast SYBR Green master mix (Qiagen), and telomere primers telg and telc, each at a final concentration of 900 nmol/L, and human β-globin primers hbgu and hbgc, each at a concentration of 500 nmol/L. Primer sequences were: telg, ACACTAAGGTTTGGGTTTGGGTTTGGGTTTGGGTTAGTGT; telc, TGTTAGGTATCCCTATCCCTATCCCTATCCCTATCCCTAACA; hbgu, CGGCGGCGGGCGGCGCGGGCTGGGCGGCTTCATCCACGTTCACCTTG; and hbgd, GCCCGGCCCGCCGCGCCCGTCCCGCCGGAGGAGAAGTCTGCCGTT. Thermal cycling conditions were as follows: Stage 1: 15 min at 95°C; Stage 2: 2 cycles of 15 sec at 94°C, and 15 sec at 49°C; and Stage 3: 32 cycles of 15 sec at 94°C, 10 sec at 62°C, 15 sec at 73°C with signal acquisition (providing cycle threshold values for the amplification of the telomere template), 10 sec at 84°C, and 15 sec at 87°C with signal acquisition (providing cycle threshold values for the amplification of the hbg template). Reactions containing serial dilutions of a reference DNA standard were included in each polymerase chain reaction plate to generate the telomere (T) and β-globin gene (S) standard curves required for quantitation. Relative mean TL, expressed as a T/S ratio, was derived as previously described (30).

### Measurement of Leukocyte TA

Leukocyte TA was measured by the Telomerase Repeat Amplification Protocol with a commercial assay (TRAPeze, Telomerase Detection Kit; Upstate/CHEMICON, Temecula, California) as described previously (9). Peripheral blood mononuclear cells were thawed at 37°C, washed twice with cold Dulbecco's phosphate buffered saline (phosphate buffered saline without magnesium and calcium) (Invitrogen, Carlsbad, California), then pelleted, and resuspended in 1 mL of Dulbecco's phosphate buffered saline. Live cells were counted with a hemacytometer (Bright-Line Hemacytometer, Reichert, Buffalo, New York) with Trypan blue (Invitrogen). One million live cells were pelleted and lysed with 1× CHAPS buffer according to the TRAPeze kit (Upstate/CHEMICON) manufacturer instructions. An extract corresponding to 5000 cells/μL was prepared for each PBMC sample, and two concentrations corresponding to 5000 and 10,000 cells were assayed for each sample to ensure that the assay was in the linear range. The reaction was performed according to the TRAPeze kit (Upstate/CHEMICON) manufacturer instructions and radioactive products fractionated by 10% polyacrylamide-8 mol/L urea sequencing gel electrophoresis. The gel was exposed to a phosphoimager plate overnight and scanned on STORM 860 (GE Healthcare). As positive control standards, 293T human cancer cells were used, and TA was expressed as the equivalent of number of 293T cells/10,000 PBMCs. The TA was quantified with ImageQuant 5.2 software (GE Healthcare) as described previously (9).

### Other Biological and Behavioral Variables

Height, weight, and waist circumference were measured by a nurse with standardized methods. Body mass index (BMI) was calculated as weight in kilograms divided by height in meters squared. Systolic (SBP) and diastolic blood pressure (DBP) were estimated from two readings obtained with a digital monitor after sitting quietly for 30 min. A nonfasting blood sample was drawn for analysis of total cholesterol, high-density lipoprotein (HDL) cholesterol, plasma triglycerides, high-sensitivity C-reactive protein (CRP), and interleukin-6 (IL-6) with methods described previously (27). Saliva was collected with a Salivette (Sarstedt, Leicester, United Kingdom), and salivary cortisol was assessed by a time-resolved immunoassay with fluorescence detection at the University of Dresden. All intra- and inter-assay coefficients of variation were <10%. Current smoking status, weekly alcohol consumption, and weekly frequency of moderate and vigorous physical exercise were assessed by questionnaire.

### Statistical Analyses

Associations between leukocyte TL and TA and between TA, TL, or cynical hostility and other demographic, biological, and behavioral variables were examined with Pearson's product moment correlations and independent samples *t* tests. Relationships between cynical hostility and TL or TA were analyzed by multiple linear regression analyses. Covariates included in initial analyses were age, gender, BMI, waist circumference, and employment grade, on the basis of evidence from the literature that these factors affect leukocyte TL and/or TA (13,15,17,31–33). Further analyses included additional blocks of covariates relating to salivary cortisol, cardiovascular measures (SBP and DBP), lipids (total and HDL cholesterol, triglycerides), inflammatory markers (IL-6, CRP), and health behaviors (smoking status, alcohol consumption, and physical activity). Results of these analyses are presented as standardized regression (β) coefficients with standard errors. Because the cynical hostility scores were somewhat skewed, analyses were repeated with square root transformation; however, results remained essentially unchanged. For illustrative purposes, analysis of variance was used to compare TL and TA in individuals belonging to low, intermediate, and high cynical hostility score tertiles, adjusting for covariates. In a second set of analyses, participants were divided into tertiles of TL and TA. We then compared participants in the lowest tertile of TL and highest tertile of TA with the remainder. Binary logistic regressions were performed to investigate the relationship between cynical hostility score and likelihood of belonging to the short TL/high TA group. Results are presented as percentages adjusted for covariates, with 95% confidence intervals (CIs).

## Results

### Participant Characteristics

Participant characteristics are presented in Table 1. The sample comprised 206 men and 228 women with a mean age of 63.3 (range 54–76) years. There was relatively equal representation from intermediate and high civil service grades, with a slightly smaller portion from low-grade employment, and the vast majority (93.8%) were nonsmokers. Participants were overweight on average with a mean BMI of 25.8. Their cynical hostility score on the CMHS indicated that levels were generally comparable with other populations, and they displayed a wide range of leukocyte TL and leukocyte TA. Participants were normotensive on average, and their salivary cortisol and plasma levels of IL-6, CRP, and lipids were within the expected range.

### Relative Leukocyte TL and TA

Leukocyte TL data were available for 434 participants, whereas TA data were available for 416 participants. Leukocyte TL was normally distributed, but TA was skewed, and hence was natural log transformed before analyses. Leukocyte TL was inversely correlated with BMI (*r* = −.102, *p* = .034) and smoking (*r* = −.090, *p* = .060, trend) and positively related to total plasma cholesterol (*r* = .099, *p* = .042). It was not related to any other demographic, biological, or behavioral variable, including age. Leukocyte TA was significantly higher in women (11.65 ± 7.4) than in men (9.66 ± 6.66) (*p* < .001) and was inversely related to blood pressure (SBP: *r* = −.111, *p* = .043, DBP: *r* = −.127, *p* = .020) and waist (*r* = −.106, *p* = .055) but not related to any other demographic, biological, or behavioral variables. There was no significant relationship between leukocyte TL and TA in men, women, or the overall sample. Compared with individuals who had missing samples, those with TL measures had similar hostility scores but were older on average (mean age 63.3 vs. 61.4 years, *p* < .001) and more likely to be female (*p* < .001) and to be from a lower employment grade (*p* < .001). There were no significant differences between characteristics of participants with TA measures versus those with missing data.

### Cynical Hostility

Scores of cynical hostility averaged 2.55 (SD 2.39). Hostility scores were significantly higher in men than in women (men 3.06 ± .17, women 2.19 ± .16, *t* [432] = 3.601, *p* < .001) and were positively related to waist (*r* = .129, *p* = .007) and CRP (*r* = .099, *p* = .041) but inversely related to employment grade (*r* = −.158, *p* = .001) and HDL cholesterol (*r* = −.111, *p* = .021). There was no relationship between hostility scores and any other demographic, biological, or behavioral measure.

### Cynical Hostility and TL

**Figure 1:**
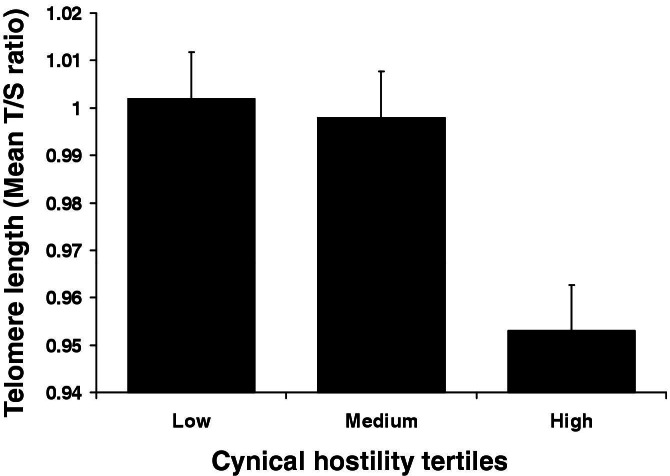
Cynical hostility and telomere length. Average leukocyte telomere length (T/S ratio) is shown for men in low-, medium-, and high-hostility tertiles. Values are adjusted for age, grade of employment, body mass index, and waist. Error bars are SEM.

Multiple linear regression analyses adjusting for age, grade, BMI, waist, and gender revealed that cynical hostility was independently associated with shorter leukocyte TL across the whole participant sample (β = −.114 [SE .049], *p* = .022). Because the gender × hostility interaction was significant (*p* = .003), men and women were analyzed separately. The relationship was highly significant in men (β = −.246 [SE .074], *p* = .001) but not in women (*p* = .442). The effect in men is illustrated in Figure 1, where the sample is divided into tertiles of hostility. Men scoring higher on cynical hostility had shorter leukocyte TL. Separate hierarchical regression analyses controlling for demographic and anthropometric variables, salivary cortisol, cardiovascular measures, plasma lipids, inflammatory markers, and health behaviors were performed to test a potential mediating role for each of these factors (Table 2). Complete information for all covariates was available for 186 of the 206 men with cynical hostility and TL data. Other factors that were independently associated with TL in the full model were plasma triglycerides (β = −.233 [SE .094], *p* = .015), plasma IL-6 (β = .192 [SE .084], *p* = .023), and alcohol consumption (β = .151 [SE .074], *p* = .044). Nevertheless, including all of the aforementioned factors in the model only modestly reduced the strength of the association between hostility and TL, as evidenced by the small changes in β coefficient, and the relationship between hostility and TL remained significant (*p* = .01). Note that CIs overlap and unstandardized regression coefficients are unchanged by inclusion of covariates in the models.

### Cynical Hostility and TA

**Figure 2:**
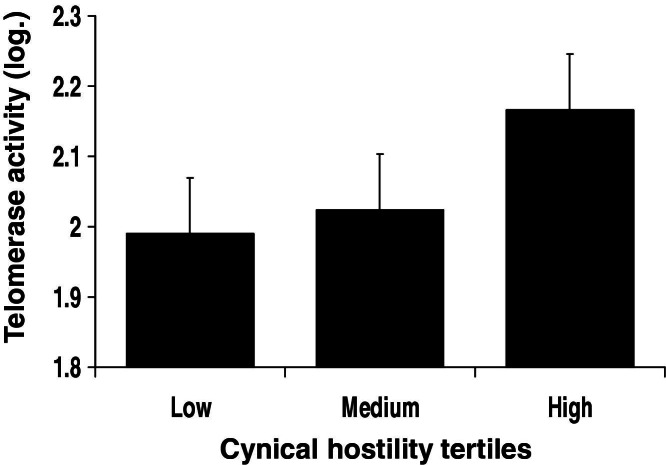
Cynical hostility and telomerase activity. Average leukocyte telomerase activity is shown for men in low-, medium-, and high-hostility tertiles. Values are adjusted for age, grade of employment, body mass index, and waist. Error bars are SEM.

Multiple linear regression analyses controlling for age, grade, BMI, waist, and gender revealed that cynical hostility was independently associated with heightened leukocyte TA (β = .095 [SE .051], *p* = .061). Because the gender × hostility interaction was significant (*p* = .033), men and women were analyzed separately. The relationship was again significant in men (β = .161 [SE .070], *p* = .022) but not in women (*p* = .823). The effect in men is illustrated in Figure 2. The sample is divided into tertiles of hostility. Men scoring higher on cynical hostility had greater TA. As in the case of TL, hierarchical regression analyses controlling for further biological and behavioral variables were performed to test a potential mediating role for each of these factors (Table 3). Complete information for all covariates was available for 216 of the 236 men with cynical hostility and TA data. No other factors were independently associated with TA in the full model, and the relationship between hostility and TA remained significant (*p* = .027).

### Cynical Hostility and Short TL Coupled with High TA

**Figure 3:**
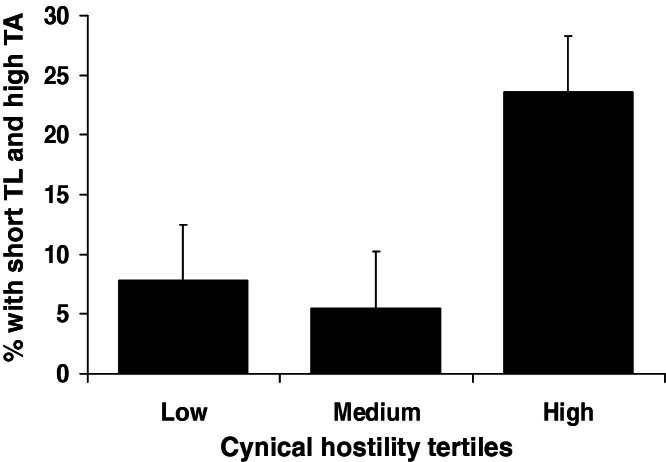
Cynical hostility and likelihood of being in the short telomere length (TL)/high telomerase activity (TA) group. The percentage of men having both short TL and high TA is shown for individuals in low-, medium-, and high-hostility tertiles. Values are adjusted for age, grade of employment, body mass index, and waist. Error bars are SEM.

The possibility that cynical hostility might be associated with the combination of short TL and high TA was tested by comparing participants in the lowest tertile of TL and highest tertile of TA with the remainder. Logistic regression analyses showed that, in men, there was a positive relationship between cynical hostility and the likelihood of being in the short TL/high TA group. As shown in Figure 3, 23.6% of men with cynical hostility scores in the highest tertile were in the short TL/high TA group, compared with 7.8% of those in the lowest hostility tertile. The odds, adjusting for age, grade, BMI, and waist, of having both short leukocyte TL and high TA were 1.28 (95% CI: 1.07–1.53, *p* = .007)/unit increase in hostility. Further supporting an interaction between cynical hostility and both TL and TA, the previously observed association of cynical hostility and TA in men (β = .161 [SE .070], *p* = .022) was attenuated when adjusting for TL (β = .073 [SE .086], *p* = .401).

## Discussion

This study provides the first evidence relating dispositional hostility and cellular aging in humans. Specifically, men scoring higher on cynical hostility had shorter leukocyte TL and heightened leukocyte TA, compared with their less-hostile counterparts. Because leukocyte telomere shortening is associated with an elevated risk of age-related disease and premature mortality (13,16,18–20), this might represent a novel physiological mechanism underlying the detrimental effects of hostility on health.

The positive association between hostility and leukocyte TA was somewhat unexpected. However, upregulated telomerase might represent a compensatory protective response to leukocyte telomere shortening in hostile individuals. Evidence suggests that telomerase offers cell protection particularly in the context of shortened telomeres. In cultured human fibroblasts, the telomerase core protein hTERT protects DNA from radiation and oxidative stress by telomere lengthening but only in cells with short telomeres (34,35). Similarly in telomerase-deficient mice models, re-introduction of telomerase preferentially restores repeats to the shortest telomeres (36,37). Prospective analyses in humans show that age-related leukocyte telomere attrition is most apparent in individuals with longer telomeres at baseline, suggesting that, as seen in animals, telomerase might specifically counteract attrition rates in individuals with short telomeres (31,38). Similar to our observations, caregivers of Alzheimer's disease patients were found to have shorter leukocyte TL but heightened leukocyte TA, compared with control subjects (8). The authors concluded that this pointed to “an unsuccessful attempt of cells to compensate for the excessive loss of TL in caregivers.” Our findings that hostile men had a significantly greater risk of having both short leukocyte TL as well as high TA, and that associations between hostility and TA were attenuated by controlling for TL, are consistent with a protective role of telomerase in hostile individuals.

The lack of association between TL and age seems contradictory to previous studies (13,17,32). Our sample had a relatively narrow age range, and participants were screened to ensure that they were generally healthy. Because leukocyte TL is associated with biological versus chronological age, it might be that a less healthy sample with a wider age range would be required to observe such an association. Notably, the majority of studies reporting an inverse association between age and TL are cross-sectional, and recent prospective evidence suggests that TL is more dynamic than previously thought and can in fact increase, remain stable, or decrease with age, depending on the individual and the environment (31,38–40).

Associations between hostility and leukocyte TL and TA were gender-specific, occurring in men only. Consistent with previous literature, cynical hostility scores were higher in men, and this might be one explanation for our findings (41). Men might also be more susceptible to the effects of stress on cellular aging as well as the detrimental consequences of aging. In a recent analysis of the Heart and Soul Study, male gender was one of the principal independent predictors of leukocyte TL shortening over 5 years (40). Similarly, in participants from the MacArthur Health Aging Study, the rate of TL shortening predicted a 2.3-fold-greater risk of 12-year cardiovascular mortality in men but not in women (39). Although there were no detectable gender differences in leukocyte TL in our participants, there is considerable evidence that TL is shorter in men than in age-matched women (31,32,42). Furthermore, men generally have a shorter life expectancy than women as well as a higher risk of premature mortality from age-related disease, particularly cardiovascular disease (32,42), and recent meta-analyses of 25 prospective studies investigating cardiovascular outcomes in initially healthy samples found that the detrimental effect of hostility on cardiovascular health was significantly greater in men (5).

A number of pathophysiological mechanisms could potentially mediate the association between hostility and cellular aging. Hostile individuals often have elevated circulating levels of inflammatory markers (IL-6, CRP) (43,44) and exaggerated inflammatory responses to acute stress (24). Inflammation triggers T-cell proliferation and enhances leukocyte turnover rate, a known cause of telomere shortening in vitro (32), and plasma levels of IL-6 and CRP were inversely correlated with leukocyte TL in humans (13,45,46). Hostile individuals often display elevated circulating levels of cortisol and catecholamines as well as heightened cortisol and catecholamine reactivity to stress (2,23,47). Cortisol reduces leukocyte TL and TA in vitro (48), and higher urinary levels of cortisol and catecholamines are associated with shorter leukocyte TL in humans (7,14). Lastly, oxidative stress accelerates leukocyte telomere shortening by preferentially damaging telomeric versus nontelomeric DNA during cell replication (32), and TA is altered under high oxidative stress (49,50). In humans, elevated urinary markers of oxidative stress were related to shorter leukocyte TL (6,17), and a recent study found a significant association between hostility and systemic oxidative stress in healthy adults (51). Because we did not measure oxidative stress or catecholamines, we cannot test these. However, accounting for circulating inflammatory markers, salivary cortisol, and cardiovascular measures as well as lipids, anthropometric, behavioral, and demographic variables did not markedly alter the relationship between cynical hostility and TL or TA in men. This suggests that, at least at basal levels, these mechanisms do not account for our findings. Nevertheless, it is possible that in a more dynamic situation, such as an acute stress response, some of these factors might play a role.

Participants in our study were all of white European origin with a relatively narrow age range and were screened to ensure that they were generally healthy. Thus our findings might not extrapolate to other population types. Analyses were cross-sectional, and we cannot draw conclusions about the causal direction of the relationship between hostility and cell aging. It is possible that cynical hostility scores and TL or TA were associated through a relationship with a third separate factor. There were some differences between individuals in the study who did and did not have TL analyzed. These were due to technical issues resulting in delay in reliable cell collection. However, hostility scores were not related to participation rates. Peripheral blood mononuclear cells are a mixed cell population, and it would be important to establish which leukocyte subtypes are involved; B cells display greater TA, whereas CD8 T cells have lower TA (52).

So far only one previous study has related a personality trait to cellular aging; O'Donovan *et al.* (46) found a strong inverse relationship between pessimism and leukocyte TL in post-menopausal women. Our findings add to this small but growing literature relating personality and cellular aging in humans, specifically highlighting a potential role for telomere attrition in mediating the detrimental effects of hostility on men's health. Although we specifically studied leukocyte TL and TA, there is evidence that TL in one tissue correlates with TL in other tissues (53). Thus, because short leukocyte TL might be a surrogate marker of senescence in the bone marrow stem cell compartment, our results might also reflect a more general relationship between hostility and organismal aging. Longitudinal studies are required to further investigate the pathways linking hostility, cellular aging, and risk of disease in humans.

## Figures and Tables

**Table 1:** Participant Characteristics

	Mean (SD) or *n* (%)	Range
Age	63.3 (5.6)	54–76
Gender		
Male	206 (47.5%)	
Female	228 (52.5%)	
Grade of Employment		
Low	112 (25.8%)	
Intermediate	167 (38.5%)	
High	155 (35.7%)	
Smoking Status		
Smoker	27 (6.2%)	
Nonsmoker	407 (93.8%)	
BMI (kg/m^2^)	25.8 (4.0)	15–43.9
Waist Circumference (cm)	85.9 (13.1)	56–125
Cynical Hostility Score	2.55 (2.39)	0–10
Telomere Length (T/S ratio)	.99 (.07)	.79–1.24
Telomerase Activity (per 1000 live cells) (*n* = 416)	10.52 (7.05)	1.23–39.98
Log Telomerase Activity (*n* = 416)	2.14 (.67)	.20–3.69
SBP (mm Hg)	123.2 (16.5)	75–189
DBP (mm Hg)	76.5 (9.5)	47–109
Salivary Cortisol (nmol/L)	6.46 (4.0)	.44–29.57
IL-6 (pg/mL)	1.36 (.84)	.30–4.98
CRP (μg/mL)	1.82 (2.42)	.048–17.88
Total Cholesterol (mmol/L)	5.3 (.93)	2.4–8.6
HDL-Cholesterol (mmol/L)	1.7 (.48)	.6–4.0
Triglycerides (mmol/L)	1.34 (.71)	.3–4.6

*N* = 434. BMI, body mass index; CRP, C-reactive protein; DBP, diastolic blood pressure; HDL, high-density lipoprotein; IL-6, interleukin-6; SBP, systolic blood pressure.

**Table 2:** Hierarchical Regression Analysis Investigating the Relationship Between Cynical Hostility and Telomere Length (Mean T/S Ratio) in Men

Model	β	SE	B	95% CI for B	*p*
1. Cynical Hostility	−.244	.071	−.007	−.011 to −.003	.001
2. Model 1 + Age, Grade, BMI, Waist	−.246	.074	−.007	−.012 to −.003	.001
3. Model 2 + Salivary Cortisol	−.240	.074	−.007	−.011 to −.003	.002
4. Model 3 + Cardiovascular Measures (SBP, DBP)	−.243	.074	−.007	−.012 to −.003	.001
5. Model 4 + Plasma Lipids (total cholesterol, HDL-cholesterol, total/HDL cholesterol ratio, triglycerides)	−.212	.074	−.006	−.011 to −.002	.005
6. Model 5 + Plasma Inflammatory Markers (IL-6, CRP)	−.219	.074	−.007	−.011 to −.002	.003
7. Model 6 + Health Behaviors (smoking, alcohol intake, physical activity)	−.193	.074	−.006	−.010 to −.001	.010

Potential mediating factors have been included as additional covariates in each model. CI, confidence interval; other abbreviations as in Table 1.

**Table 3:** Hierarchical Regression Analysis Investigating the Relationship Between Cynical Hostility and Telomerase Activity in Men

Model	β	SE	B	95% CI for B	*p*
1. Cynical Hostility	.169	.067	.045	.010–.081	.013
2. Model 1 + Age, Grade, BMI, Waist	.161	.070	.043	.006–.080	.022
3. Model 2 + Salivary Cortisol	.167	.070	.045	.008–.082	.018
4. Model 3 + Cardiovascular Measures (SBP, DBP)	.163	.071	.044	.006–.081	.022
5. Model 4 + Plasma Lipids (total cholesterol, HDL-cholesterol, total/HDL cholesterol ratio, triglycerides)	.173	.071	.046	.009–.084	.015
6. Model 5 + Plasma Inflammatory Markers (IL-6, CRP)	.169	.071	.046	.008–.083	.017
7. Model 6 + Health Behaviors (smoking, alcohol intake, physical activity)	.161	.072	.043	.005–.081	.027

Potential mediating factors have been included as additional covariates in each model. Abbreviations as in Tables 1 and 2.
